# The Prognostic Value of Frailty Assessment Tools in Predicting Postoperative Outcomes After Revision Total Hip and Knee Arthroplasty: A Systematic Review

**DOI:** 10.3390/jcm15124489

**Published:** 2026-06-10

**Authors:** Ruben David Braescu, Jenel Marian Pătrașcu, Jenel Marian Pătrașcu, Dan Grigore Cojocaru

**Affiliations:** 1 Doctoral School, “Victor Babes” University of Medicine and Pharmacy, Eftimie Murgu Square 2, 300041 Timisoara, Romania; rubendavidbraescu@yahoo.com; 2 “Pius Brinzeu” Emergency Clinical County Hospital, Bld. Liviu Rebreanu No. 156, 300723 Timisoara, Romania; dr_cojocaru@yahoo.com; 3 Department XV—Discipline of Orthopedics—Traumatology, “Victor Babes” University of Medicine and Pharmacy, Eftimie Murgu Square No. 2, 300041 Timisoara, Romania; 4 Research Center University Professor Doctor Teodor Șora, “Victor Babes” University of Medicine and Pharmacy, Eftimie Murgu Square No. 2, 300041 Timisoara, Romania

**Keywords:** frailty, revision total joint arthroplasty, revision total hip arthroplasty, revision total knee arthroplasty, postoperative complications, prognostic factors, modified frailty index, hospital frailty risk score, systematic review

## Abstract

**Background/Objectives:** Frailty has emerged as a relevant marker of biological vulnerability in patients undergoing complex orthopedic procedures, yet its specific prognostic value in revision total hip and knee arthroplasty remains incompletely synthesized. This systematic review evaluated whether validated preoperative frailty assessment tools are associated with adverse postoperative outcomes after revision total joint arthroplasty and whether available studies allow comparison of prediction performance across instruments. **Methods:** A systematic search of PubMed/MEDLINE, Embase, the Cochrane Library, Web of Science, Scopus, citation lists, and selected gray-literature sources was performed from inception through January 2026. Gray-literature records and conference abstracts were used only for citation tracking; the synthesis included only full-length peer-reviewed original research articles involving adult patients undergoing revision total hip arthroplasty, revision total knee arthroplasty, or both, with quantitative outcomes according to a validated frailty measure. Because of heterogeneity in frailty tools, outcome definitions, revision indications, and adjustment strategies, findings were synthesized narratively and certainty was assessed by outcome domain. **Results:** Eleven full-length studies were included, with cohorts ranging from 117 patients to 576,920 admissions, and most were retrospective database analyses. Higher frailty burden was consistently associated with worse short-term outcomes, including complications, prolonged hospital stay, readmission, non-home discharge, resource use, and mortality-related risk stratification. Representative findings included 30-day readmission of 23.8% versus 9.9%, surgical complications of 28.6% versus 7.8%, and odds ratios of up to 10.79 for complications across escalating frailty strata. Prediction studies suggested stronger discrimination for revision-specific or broader models, such as CARDE-B, RAI-rev, and machine-learning approaches, than for simpler generic frailty indices. **Conclusions:** Frailty is a consistent preoperative marker of elevated short-term perioperative risk after revision arthroplasty. The available evidence supports incorporating frailty assessment into preoperative risk stratification and counseling, but it remains insufficient to establish one universally preferred tool or to prove that frailty screening alone improves outcomes without targeted intervention.

## 1. Introduction

Total joint arthroplasty (TJA), encompassing total hip arthroplasty (THA) and total knee arthroplasty (TKA), represents one of the most successful and commonly performed surgical interventions in modern orthopedic practice [1]. As the global population ages and life expectancy continues to increase, the demand for primary and revision TJA procedures has risen substantially over the past two decades [2]. In the United States alone, projections estimate that the number of revision THA and TKA procedures will increase by more than 130% and 190%, respectively, by the year 2030, placing enormous strain on healthcare resources and requiring increasingly sophisticated preoperative risk stratification tools [3]. Revision arthroplasty, defined as the partial or complete replacement of a previously implanted prosthetic joint, is inherently more complex than primary procedures and is associated with significantly higher rates of perioperative complications, prolonged hospital stays, increased healthcare expenditures, and diminished patient-reported functional outcomes [4]. The complexity of revision surgery arises from multiple factors, including compromised bone stock, altered soft tissue envelopes, the presence of infection, and the need for more constrained implant designs, all of which contribute to a complication rate that is two to three times higher than that observed following primary TJA [5].

The postoperative outcomes following revision TJA are considerably less predictable than those observed after primary arthroplasty, with reported complication rates ranging from 15% to 35% depending on the indication for revision and the patient population studied [6]. Common complications include periprosthetic joint infection, periprosthetic fracture, instability, aseptic loosening, wound healing problems, thromboembolic events, and medical complications such as cardiac events, pneumonia, and urinary tract infections [7]. Furthermore, revision TJA is associated with a 30-day mortality rate of 0.5% to 2.5%, which is considerably higher than the 0.1% to 0.4% mortality rate observed after primary procedures [8]. The economic burden of revision arthroplasty is also substantial, with mean hospital costs for revision THA and TKA estimated at approximately $25,000 to $75,000 per case, depending on the complexity and indication for surgery [9]. Given the anticipated increase in revision TJA volume and the substantial clinical and economic implications of postoperative complications, there is a critical need for reliable preoperative risk assessment tools that can accurately identify patients at elevated risk for adverse outcomes, thereby enabling targeted perioperative optimization strategies and informed shared decision-making [10].

Frailty is a multidimensional geriatric syndrome characterized by decreased physiological reserve and increased vulnerability to stressors, resulting in an impaired ability to maintain homeostasis following a physiological insult such as surgery [11]. Unlike individual comorbidity measures or chronological age alone, frailty captures a state of accumulated deficits across multiple organ systems and functional domains, providing a more comprehensive assessment of a patient’s biological vulnerability [12]. The concept of frailty has gained increasing recognition in the surgical literature as a powerful prognostic indicator that transcends traditional risk factors, such as age, body mass index, and American Society of Anesthesiologists (ASA) classification [13]. In the context of orthopedic surgery, frailty has been shown to predict adverse outcomes, including postoperative complications, extended length of hospital stay, unplanned readmissions, discharge to skilled nursing facilities, and short-term mortality following a wide range of musculoskeletal procedures [14]. The prevalence of frailty among patients presenting for revision TJA is estimated to be between 15% and 30%, which is notably higher than the 8% to 20% prevalence observed in primary arthroplasty cohorts, reflecting the older age, greater comorbidity burden, and diminished functional status that characterize the revision population [15].

Multiple frailty assessment instruments have been developed and validated for surgical or acute-care populations, each based on different conceptual frameworks and assessment domains. Commonly used instruments include deficit-accumulation indices, such as the Modified Frailty Index (mFI), administrative tools, such as the Hospital Frailty Risk Score (HFRS), clinician-rated tools, such as the Clinical Frailty Scale (CFS), and revision-specific prediction scores, such as CARDE-B. These tools differ in feasibility, data requirements, thresholds, and target outcomes; therefore, their results should not be interpreted as interchangeable measures of the same construct.

Although frailty has been examined in primary or mixed total joint arthroplasty populations, the revision arthroplasty evidence base remains fragmented and heterogeneous. Existing studies differ in revision indication, frailty instrument, outcome window, sample size, and statistical adjustment strategy, which limits direct comparison and precludes a single pooled estimate across outcomes. This knowledge gap is clinically important because revision THA and revision TKA patients represent a vulnerable population in whom risk stratification may influence perioperative planning, discharge preparation, resource allocation, and shared decision-making.

The primary objective of this systematic review was to evaluate and synthesize the current evidence on the prognostic value of preoperative frailty assessment tools in predicting postoperative complications, readmission, discharge disposition, resource use, and mortality after revision THA and TKA. Specifically, this review aimed to: (1) identify full-length peer-reviewed studies evaluating validated frailty instruments in revision arthroplasty cohorts; (2) characterize the instruments, thresholds, study periods, follow-up windows, and adjusted covariates used; (3) compare predictive performance descriptively by separating association measures, such as odds ratios or incidence contrasts, from model-discrimination measures, such as AUC or C-statistics; and (4) identify evidence gaps requiring prospective, head-to-head, and interventional study designs.

## 2. Materials and Methods

### 2.1. Study Design and Protocol Registration

This systematic review was conducted in accordance with the Preferred Reporting Items for Systematic Reviews and Meta-Analyses (PRISMA) 2020 guidelines and structured using the Population, Intervention/Prognostic factor, Comparator, Outcome, and Study Design (PICOS) framework. A protocol was developed a priori but was not registered in a public registry such as PROSPERO, OSF, or INPLASY; this is acknowledged as a limitation. The population of interest was adult patients undergoing revision total hip arthroplasty (rTHA), revision total knee arthroplasty (rTKA), or both; the prognostic factor was preoperative frailty measured using a validated tool; the comparator was a non-frail or lower-frailty group within the same cohort; and eligible outcomes included complications, readmission, mortality, length of stay, infection-related outcomes, reoperation, resource use, and discharge disposition.

### 2.2. Search Strategy and Information Sources

A comprehensive search was performed in PubMed/MEDLINE, Embase, the Cochrane Library, Web of Science Core Collection, and Scopus from database inception through January 2026. The strategy combined controlled vocabulary and free-text terms for revision arthroplasty, frailty instruments, and postoperative outcomes. No language restrictions were applied at the search stage, and the complete database-specific search strings are provided in Appendix A. Reference lists of included studies and relevant reviews were manually screened. Gray-literature searching included American Academy of Orthopedic Surgeons (AAOS), European Federation of National Associations of Orthopedics and Traumatology (EFORT), and International Society of Arthroplasty Registries (ISAR) conference proceedings from 2019 through January 2026; conference records were used only for citation tracking and were not treated as eligible evidence unless a corresponding full-length peer-reviewed article was available.

### 2.3. Eligibility Criteria and Study Selection

Two reviewers independently screened titles and abstracts, retrieved potentially eligible full texts, and determined final eligibility using predefined criteria. Studies were included if they: (1) enrolled adults undergoing rTHA, rTKA, or both; (2) used a validated and reproducible frailty assessment instrument as a preoperative prognostic factor; (3) reported quantitative revision-specific associations with at least one postoperative outcome; and (4) were published as full-length original research articles in peer-reviewed journals. Studies were excluded if they evaluated only primary arthroplasty without a separate revision analysis, used non-validated or ad hoc frailty definitions, reported only conference-abstract data without a peer-reviewed full article, were reviews, editorials, or commentaries, or addressed revision shoulder, ankle, or elbow arthroplasty. Disagreements were resolved by discussion, with adjudication by a third reviewer when needed.

### 2.4. Data Extraction and Risk-of-Bias Assessment

A standardized extraction form was pilot-tested and then applied independently by two reviewers. Extracted variables included author, year, country, design, data source, study period, revision type, revision indication, sample size, follow-up duration, frailty tool and threshold, adjustment variables, and all reported quantitative outcome estimates. All included studies were full-length peer-reviewed articles. When a full article did not report a descriptive variable or outcome detail, the field was recorded as not reported; no missing percentages, cohort descriptors, or effect estimates were imputed from conference-only abstracts. Methodological quality was assessed with the Newcastle–Ottawa Scale for observational studies, and the risk-of-bias assessment was considered when interpreting each frailty-outcome association.

### 2.5. Data Synthesis and Certainty of Evidence

Because of heterogeneity in frailty tools, thresholds, revision indications, outcome definitions, follow-up windows, and adjustment strategies, a narrative synthesis was performed rather than a formal meta-analysis. The synthesis followed Synthesis Without Meta-analysis principles and was organized by frailty instrument, outcome domain, and metric type. Association metrics, including odds ratios, incidence contrasts, hazard ratios, and risk ratios, were summarized separately from model-performance metrics, such as AUC, C-statistics, and Brier scores, because these quantities are not directly comparable on a single scale. Certainty of evidence was assessed by outcome domain using a modified GRADE framework for prognostic-factor evidence, considering study quality, consistency, precision, indirectness, and potential publication bias.

## 3. Results

Eleven full-length peer-reviewed studies met the eligibility criteria and were included in the qualitative synthesis and quantitative tabulation. The included evidence was clinically and methodologically heterogeneous, with mixed revision THA/TKA cohorts, isolated revision THA and revision TKA cohorts, septic revision series, and two-stage revision studies; therefore, results are presented narratively and in tables rather than as pooled meta-analytic estimates.

### 3.1. Study Selection

**Figure 1:**
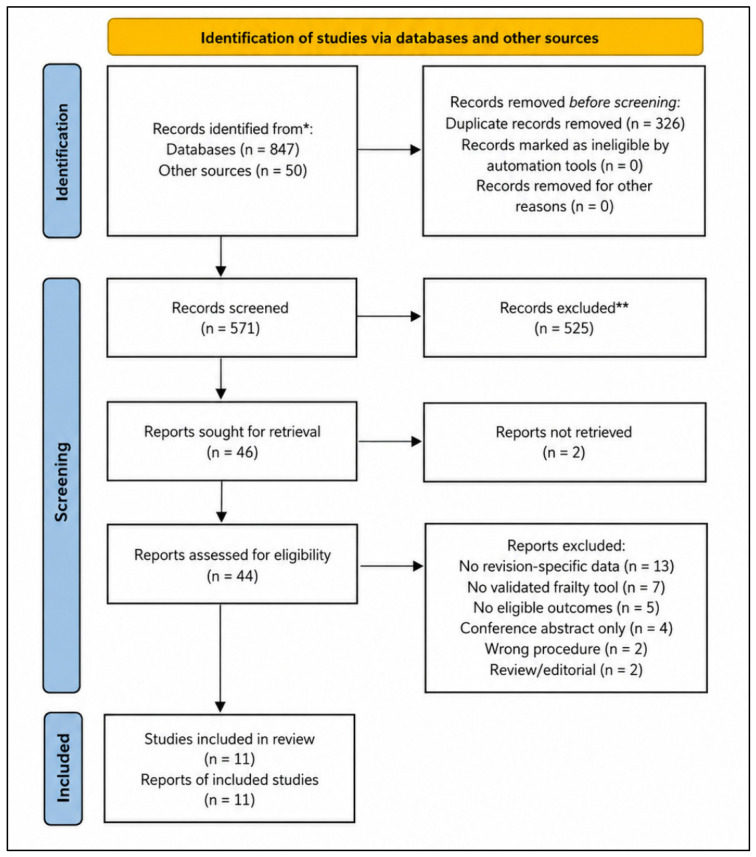
PRISMA 2020 study-selection flow diagram; * Databases used: PubMed/MEDLINE, Embase, the Cochrane Library, Web of Science, Scopus; ** Excluded for not matching the study topic.

Figure 1 summarizes the PRISMA 2020 study-selection pathway. Database searches identified 847 records and additional sources identified 50 records. After duplicate removal, 571 records were screened by title and abstract, of which 525 were excluded. Forty-six reports were sought for retrieval, two were not retrieved, and 44 full-text reports were assessed for eligibility. Thirty-three reports were excluded for absence of revision-specific data, lack of a validated frailty tool, no eligible outcomes, conference-abstract-only status, wrong procedure, or review/editorial design. Eleven full-length peer-reviewed studies were included in the final synthesis.

### 3.2. Characteristics of the Included Studies

Table 1 shows that the evidence base is dominated by retrospective studies [16,17,18,19,20,21,22,23,24,25,26], particularly large U.S. administrative datasets, with publication years spanning 2019 to 2025. The largest cohort was the revision TKA inpatient analysis by Arapovic et al. [24] with 576,920 patients, followed by Kyaw et al. [23] with 47,347 revision TKA cases, Tram et al. [22] with 36,243 revision THA cases, and Zamanzadeh et al. [19] with 32,069 aseptic revision THA/TKA procedures. Frailty assessment was most commonly performed with mFI-derived tools and HFRS, although CARDE-B [18], ICD-9 frailty coding [24], and RAI-rev [26] were also represented. Where demographic data were available, mean or median age clustered in the mid-to-late 60s, and women accounted for 46.4% to 58.9% of participants, indicating that the included populations were predominantly older adults undergoing high-risk revision procedures.

### 3.3. Quantitative Findings Across Studies

Table 2 demonstrates a highly consistent pattern: greater frailty was associated with worse postoperative outcomes across nearly every domain assessed. Particularly notable findings included 30-day readmission of 23.8% versus 9.9% and surgical complications of 28.6% versus 7.8% in intermediate/high versus low HFRS groups in Meyer et al. [17], with an odds ratio of 3.45 for surgical complications. Zamanzadeh et al. [19] showed a dose–response increase in any 30-day complication from 15% to 45% in revision THA and from 5% to 55% in revision TKA across increasing aamFI categories, while Shi et al. [20] found that the malnourished–frail group had odds ratios of 3.71 for reinfection, 4.81 for complications, 4.91 for 60-day readmission, and 5.78 for prolonged stay. In revision THA, Momtaz et al. [21] reported that the odds of any complication rose from 1.43 to 3.17 to 10.79 across escalating frailty strata, with corresponding readmission odds ratios of 1.45, 2.50, and 4.10. Mortality prediction also improved with more tailored models, including CARDE-B with an AUC of 0.85 versus 0.77 for ASA and 0.67 for mFI-5 in Raad et al. [18], and machine-learning models with AUCs of 0.93–0.94 in Pean et al. [25].

### 3.4. Outcome Domains and Evidence Quality

**Figure 2:**
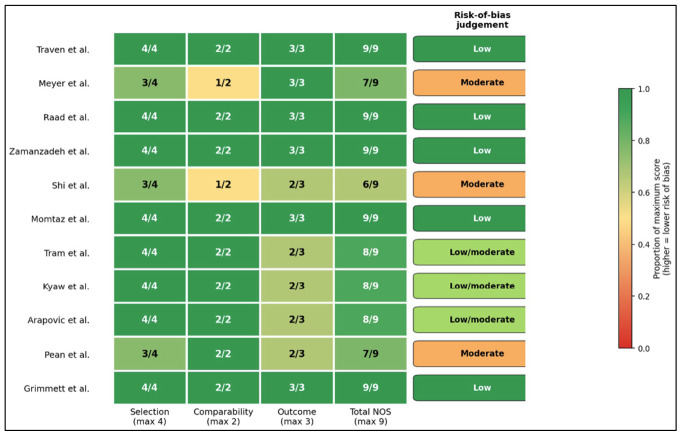
Newcastle–Ottawa Scale risk-of-bias assessment for included observational studies [16,17,18,19,20,21,22,23,24,25,26].

The risk-of-bias assessment indicated that most large database studies had low or low-to-moderate risk of bias, while smaller single-center cohorts and prediction reports with incomplete descriptive data were interpreted more cautiously. These judgements did not change the direction of the synthesis but reduced the certainty assigned to sparse or highly heterogeneous outcome domains (Figure 2).

**Figure 3:**
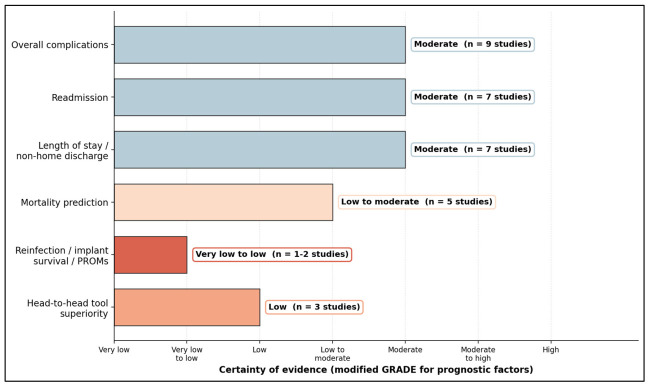
Outcome domains reported across included studies.

Figure 3 highlights that the revision–frailty literature is concentrated mainly on short-term perioperative outcomes rather than long-term revision-specific endpoints. The most consistently reported domains were postoperative complications, length of stay, readmission, discharge disposition, and mortality, which appeared repeatedly across the included studies (Table 3). By contrast, more specialized endpoints, such as transfusion, hospital cost, reoperation, and reinfection, were examined in smaller subsets of studies, such as Meyer et al. [17] for transfusion, Shi et al. [20] for reinfection, and Tram et al. [22] and Kyaw et al. [23] for cost and reoperation.

**Figure 4:**
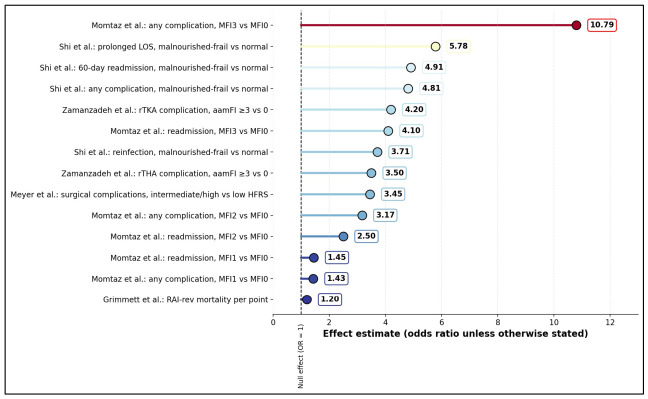
Selected association effect estimates showing higher risk with greater frailty burden. Values shown are effect estimates, primarily odds ratios, and do not include AUC or C-statistic values [16,17,18,19,20,21,22,23,24,25,26].

Figure 4 captures selected association effect estimates and separates them from model-discrimination statistics. The steepest gradient was reported by Momtaz et al. [21], where the odds of any complication increased from 1.43 in MFI1 to 3.17 in MFI2 and 10.79 in MFI3 relative to MFI0, with corresponding readmission odds ratios of 1.45, 2.50, and 4.10. Shi et al. [20] reported elevated risks in malnourished–frail patients, including OR 5.78 for prolonged length of stay, OR 4.91 for 60-day readmission, OR 4.81 for any complication, and OR 3.71 for reinfection. Zamanzadeh et al. [19] and Meyer et al. [17] also showed higher complication risk among frailer patients. These estimates support a graded association between frailty burden and adverse early outcomes, but they should not be interpreted as interchangeable with prediction-model AUC values.

**Figure 5:**
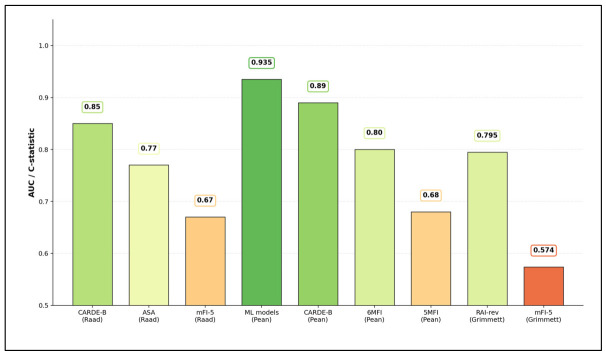
Model-discrimination metrics reported in studies directly comparing frailty-related tools.

Figure 5 displays model-discrimination metrics from studies that directly compared prediction tools. CARDE-B outperformed ASA and mFI-5 for 30-day mortality prediction in Raad et al. [18], machine-learning models outperformed CARDE-B and modified frailty indices in Pean et al. [25], and RAI-rev outperformed mFI-5 in septic revision arthroplasty in Grimmett et al. [26]. These findings suggest that broader or revision-specific models may improve discrimination for selected endpoints, but the small number of direct comparisons prevents a universal recommendation for one instrument.

## 4. Discussion

### 4.1. Analysis of Findings

This systematic review indicates that frailty is consistently associated with worse early outcomes after revision arthroplasty, but the findings should be interpreted primarily as evidence of association and risk prediction rather than proof of clinical utility. Across the included cohorts, patients with higher frailty burden experienced more postoperative complications, longer hospitalization, greater likelihood of readmission, more frequent non-home discharge, higher transfusion use, increased cost, and a higher predicted risk of short-term mortality when frailty was incorporated into risk-prediction models [16,17,18,19,20,21,22,23,24,25,26]. The association was observed in hip and knee revision settings, general revision datasets, indication-specific cohorts, and both administrative and institutional series. However, heterogeneity in revision indication, infection status, joint type, outcome windows, and covariate adjustment prevents pooled conclusions about the absolute magnitude of risk for a single standardized revision pathway.

A second major observation is that multiple frailty instruments appear informative, but the current evidence does not justify declaring a universally superior tool for all revision contexts. mFI-based tools, HFRS, CARDE-B, ICD-based frailty coding, and RAI-rev each captured clinically meaningful postoperative risk in at least one revision population [16,17,18,19,20,21,22,23,24,25,26]. However, relatively few studies directly compared instruments within the same cohort. Where such comparisons were performed, the results suggest that revision-specific or broader multidimensional models may outperform simpler indices for selected endpoints. CARDE-B improved mortality discrimination over ASA class and mFI-5 after revision TJA, RAI-rev outperformed mFI-5 in septic revision arthroplasty, and machine-learning models further improved mortality prediction in a separate revision cohort [18,25,26,27]. These findings support tailored tool selection based on the clinical setting, available data infrastructure, and the outcome of interest rather than assuming interchangeability among frailty measures.

The findings have practical implications for perioperative care, while remaining hypothesis-generating. Frailty screening may be most useful as a trigger for structured multidisciplinary review rather than as an isolated exclusion criterion. In clinical practice, frail revision candidates could be considered for nutrition assessment and protein/calorie optimization, anemia and glycemic optimization, medication review, fall-risk and functional assessment, prehabilitation or supervised conditioning where time allows, early social-work involvement, discharge planning, and closer postoperative monitoring. In septic or two-stage revision pathways, frailty assessment may also help identify patients requiring coordinated infectious-disease, nutrition, rehabilitation, and wound-care input [28,29,30,31,32,33,34]. Nevertheless, the present evidence does not demonstrate that any single frailty tool should be universally adopted or that screening alone improves outcomes without an actionable optimization pathway.

Several priorities emerge for future research. First, studies should report revision-specific effect estimates and avoid pooling primary and revision arthroplasty unless separate subgroup results are provided. Second, direct head-to-head comparisons of HFRS, mFI-5, aamFI, CARDE-B, RAI-rev, CFS, and other clinician-rated instruments are needed within the same aseptic, septic, hip, and knee revision cohorts. Third, long-term revision-specific endpoints should be incorporated, including implant survival, reinfection-free survival, reoperation, functional recovery, and PROMs. Fourth, prospective interventional studies should test whether frailty-informed care pathways modify risk. Feasible designs include randomized or stepped-wedge trials of nutrition optimization, multimodal prehabilitation, anemia correction, medication optimization, enhanced postoperative monitoring, and structured discharge planning for patients exceeding predefined frailty thresholds. Nevertheless, these findings should be interpreted in light of potential residual confounding from unmeasured or incompletely controlled factors, including underlying comorbidities and other patient- and treatment-related characteristics [35,36,37,38,39,40,41,42,43].

### 4.2. Study Limitations

This review has several limitations. All included studies were observational and most were retrospective database analyses, limiting causal inference and creating dependence on coding accuracy and variable availability. The evidence base mixed aseptic and septic revisions, revision THA and revision TKA, single-center cohorts and national datasets, and varying outcome definitions and follow-up windows. Most outcomes were short term, frequently 30 days, whereas implant survival, reinfection-free survival, and patient-reported recovery were rarely assessed. Few studies directly compared frailty tools within the same cohort, and most studies originated from the United States, limiting generalizability to other health systems, discharge structures, and perioperative pathways. In addition, although the review protocol was prepared a priori, it was not registered in a public registry, which may limit transparency and reproducibility. These limitations reduced the certainty of evidence and support cautious interpretation of tool superiority and clinical implementation claims.

## 5. Conclusions

The available revision-specific literature supports frailty as an important preoperative marker of elevated short-term perioperative risk after revision THA and TKA. Across diverse cohorts and frailty instruments, higher frailty was associated with more complications, longer hospital stay, greater readmission risk, more non-home discharge, increased resource use, and improved discrimination of short-term mortality when frailty variables were incorporated into revision-specific or broader prediction models rather than used as stand-alone indices. These findings support considering frailty assessment during preoperative evaluation and patient counseling for revision arthroplasty. However, because the evidence is predominantly retrospective and heterogeneous, it remains insufficient to identify one universally superior instrument or to establish that frailty screening improves outcomes unless linked to targeted perioperative optimization.

## Figures and Tables

**Table 1:** Characteristics of the included studies and directly reported cohort descriptors.

Study	Country; Data Source	Design; Study Period	Revision Cohort	N	Frailty Tool/Threshold	Follow-Up	Adjustment Variables/Model	Main Outcomes	Key Revision-Specific Findings
Traven et al. [16]	USA; ACS-NSQIP	Retrospective database; 2006–2015	rTHA + rTKA	30,252	mFI-5; increasing score categories	30 days	Multivariable models using demographic, comorbidity, and procedural variables available in NSQIP	Serious medical complications, LOS, facility discharge, readmission, mortality	mFI-5 independently predicted serious medical complications, prolonged stay, non-home discharge, readmission, and mortality.
Meyer et al. [17]	Germany; single center	Retrospective cohort; 2013–2019	rTHA + rTKA	565	HFRS; low vs. intermediate/high risk	30 days	Regression models adjusted for baseline clinical and surgical variables reported by the authors	Readmission, complications, transfusion	Readmission was 23.8% vs. 9.9% and surgical complications 28.6% vs. 7.8%; OR 3.45 for surgical complications.
Raad et al. [18]	USA; ACS-NSQIP with NIS validation	Retrospective derivation/validation; 2005–2016	Revision TJA	13,118 derivation; 19,153 validation	CARDE-B score; compared with ASA and mFI-5	30 days	Revision-specific mortality prediction model with external validation	30-day mortality discrimination	CARDE-B AUC 0.85 in derivation and 0.75 in validation, outperforming ASA and mFI-5.
Zamanzadeh et al. [19]	USA; national database	Retrospective database; 2015–2020	Aseptic rTHA + rTKA	32,069	Age-adjusted mFI (aamFI); categories 0 to >=5; age >= 73 years as one component	30 days	Multivariable models adjusted for demographics, comorbidities, and operative characteristics	Any 30-day complication, mortality	Complication incidence increased from 15% to 45% in rTHA and 5% to 55% in rTKA across frailty strata.
Shi et al. [20]	China; single center	Retrospective cohort; 2010–2020	Two-stage revision for chronic PJI	117	mFI-11 plus albumin-defined malnutrition; combined frailty/malnutrition groups	60 days and infection follow-up reported by authors	Multivariable models including nutrition and clinical variables	Reinfection, complications, readmission, prolonged LOS	Malnourished–frail patients had OR 3.71 for reinfection, 4.81 for complications, 4.91 for 60-day readmission, and 5.78 for prolonged stay.
Momtaz et al. [21]	USA; ACS-NSQIP	Retrospective database; 2015–2020	Revision THA	17,868	Custom 8-item MFI; MFI0-MFI3	30 days	Multivariable models adjusted for demographic and comorbidity factors	Complications, readmission, mortality, discharge	Dose–response: any-complication ORs 1.43, 3.17, and 10.79 across MFI1-MFI3; readmission ORs 1.45, 2.50, and 4.10.
Tram et al. [22]	USA; NRD	Retrospective national readmission database; 2016–2019	Revision THA	36,243	HFRS; frail vs. non-frail	30 days/index admission	Models adjusted for demographic, payer, hospital, and comorbidity variables	Readmission, LOS, cost, complications, reoperation	Frailty was associated with higher readmission, longer stay, greater costs, more complications, and more reoperation.
Kyaw et al. [23]	USA; NRD	Retrospective national readmission database; 2016–2019	Revision TKA	47,347	HFRS; frail vs. non-frail	30 days/index admission	Models adjusted for demographic, hospital, and comorbidity variables	Readmission, LOS, cost, complications, reoperation	Frailty remained prognostic across loosening, infection, and instability indications; infection readmission 13.5% vs. 8.1%.
Arapovic et al. [24]	USA; NIS	Retrospective inpatient database; 2005–2014	Revision TKA	576,920	ICD-9 frailty coding; frail vs. non-frail	Index hospitalization	Propensity score-weighted analysis	In-hospital complications, discharge, LOS	Frailty was associated with postoperative complications, non-home discharge, and longer hospitalization.
Pean et al. [25]	USA; ACS-NSQIP	Retrospective database; 2005–2020	Revision TJA	Not separately stated in accessible full-text record	CARDE-B, 5MFI, 6MFI and machine-learning models	30 days	Machine-learning and comparative prediction models	Mortality prediction	ML models achieved AUC 0.93–0.94, exceeding CARDE-B, 6MFI, and 5MFI.
Grimmett et al. [26]	USA; ACS-NSQIP	Retrospective database; 2008–2021	Septic revision THA + TKA	4395	RAI-rev vs. mFI-5	30 days	Comparative C-statistic models	Mortality and non-home discharge prediction	RAI-rev outperformed mFI-5 for mortality C-statistic (0.795 vs. 0.574) and non-home discharge (0.670 vs. 0.602).

**Table 2:** Key findings across the included studies.

Clinical Interpretation	Outcome Domain	Comparison/Frailty Definition	Key Quantitative Findings	Study
This large ACS-NSQIP study supports routine frailty stratification even when only a short administrative index is available.	Complications/LOS/readmission/discharge/mortality	mFI-5 modeled as a preoperative frailty score	Frailty independently predicted serious medical complications, discharge to a facility, longer stay, readmission, and mortality after revision THA/TKA.	Traven et al. [16]
Higher HFRS identified a small but distinctly high-risk subgroup with substantially worse early outcomes.	Readmission/complications/transfusion	Intermediate-high vs. low HFRS	30-day readmission 23.8% vs. 9.9%; surgical complications 28.6% vs. 7.8%; OR 3.45 (95% CI 1.45–8.18) for surgical complications.	Meyer et al. [17]
Revision-specific risk modeling can improve discrimination beyond general perioperative or generic frailty tools.	Mortality prediction	CARDE-B vs. ASA vs. mFI-5	30-day mortality 0.7%; AUC 0.85 for CARDE-B versus 0.77 for ASA and 0.67 for mFI-5 in derivation, with AUC 0.75 in external validation.	Raad et al. [18]
The age-adjusted index showed a clear dose–response pattern across both major revision arthroplasty settings.	Any 30-day complication	aamFI 0 to >=5; reference aamFI 0	Any-complication incidence rose from 15% to 45% in rTHA and from 5% to 55% in rTKA across aamFI categories; aamFI >= 3 gave OR 3.5 in rTHA and OR 4.2 in rTKA.	Zamanzadeh et al. [19]
Frailty appears especially consequential when combined with poor nutritional reserve in chronic PJI revision pathways.	Reinfection/complications/LOS/readmission	Combined malnutrition + frailty vs. normal nutrition and non-frailty	Compared with the normal group, the malnourished–frail group had OR 3.71 for reinfection, OR 4.81 for complications, OR 4.91 for 60-day readmission, and OR 5.78 for prolonged stay.	Shi et al. [20]
The steep gradient across frailty strata suggests a clinically meaningful accumulation-of-deficits effect in revision THA.	Complications/readmission/mortality/discharge	Increasing custom MFI burden (MFI1 to MFI3) vs. MFI0	Relative to MFI0, odds of any complication were 1.43, 3.17, and 10.79 for MFI1, MFI2, and MFI3; corresponding readmission ORs were 1.45, 2.50, and 4.10.	Momtaz et al. [21]
Administrative frailty scoring remained informative across large national revision THA cohorts and multiple indications.	Readmission/LOS/cost/complications/reoperation	Frail vs. non-frail by HFRS	Across revision THA indications, frailty was associated with higher 30-day readmission, longer stay, greater cost, more complications, and more reoperation; in dislocation revisions, ORs reached 1.96 for readmission and 1.85 for longer stay.	Tram et al. [22]
Frailty carried prognostic value regardless of whether revision TKA was performed for aseptic or septic reasons.	Readmission/LOS/cost/complications/reoperation	Frail vs. non-frail by HFRS	In revision TKA for loosening, readmission was 7.8% vs. 3.7% and complications 6.8% vs. 2.9%; in infection, readmission was 13.5% vs. 8.1% and complications 14.0% vs. 8.3%; in instability, readmission was 8.7% vs. 3.9% and complications 8.0% vs. 3.5%.	Kyaw et al. [23]
A population-level inpatient analysis confirmed that frailty is already clinically visible during the index hospitalization.	In-hospital complications/discharge/LOS	Frail vs. non-frail by ICD-9 frailty coding	Frail revision TKA recipients had higher in-hospital postoperative complications, including DVT, postoperative anemia, respiratory complications, and wound dehiscence, with lower home discharge rates and longer stay.	Arapovic et al. [24]
Prediction performance may improve when frailty-related variables are embedded in broader nonlinear risk models.	Mortality prediction	ML models vs. CARDE-B vs. 6MFI vs. 5MFI	Machine-learning models reached AUC 0.93–0.94 and Brier score 0.005 for 30-day mortality, outperforming CARDE-B (0.89), 6MFI (0.80), and 5MFI (0.68).	Pean et al. [25]
Tool selection matters, particularly in septic revision cohorts with substantial physiological stress and complex discharge needs.	Mortality/discharge prediction	RAI-rev vs. mFI-5	RAI-rev outperformed mFI-5 for mortality discrimination (C-statistic 0.795 vs. 0.574) and non-home discharge (0.670 vs. 0.602) in septic revision arthroplasty.	Grimmett et al. [26]

**Table 3:** Certainty-of-evidence assessment by outcome domain using a modified GRADE framework for prognostic-factor studies.

Outcome Domain	No. Studies	Evidence Base	Consistency	Key Limitations	Certainty	Conclusion Supported
Overall complications	9	Retrospective observational cohorts and databases	Consistently worse outcomes with higher frailty	Heterogeneous definitions, mixed hip/knee and septic/aseptic revisions	Moderate	Frailty is a reliable marker of elevated short-term complication risk.
Readmission	7	Mostly 30-day administrative outcomes	Generally consistent increased risk	Variable readmission windows and adjustment variables	Moderate	Frailty is associated with higher early readmission.
Length of stay/non-home discharge	7	Database and institutional cohorts	Consistent direction of effect	Discharge practices differ by health system	Moderate	Frailty informs discharge planning and resource use.
Mortality prediction	5	Prediction model and comparative-tool studies	Consistent improvement with tailored models	Low event rates and retrospective design	Low to moderate	Frailty-related models improve short-term mortality discrimination, but clinical thresholds remain unresolved.
Reinfection/implant survival/patient-reported outcome measures (PROMs)	1–2	Sparse revision-specific evidence	Insufficient for firm conclusions	Few studies, limited follow-up	Very low to low	Long-term revision-specific endpoints require prospective study.
Head-to-head tool superiority	3	Direct comparisons of CARDE-B, mFI, ML models and RAI-rev	Suggests tailored tools may perform better	Few same-cohort comparisons and differing endpoints	Low	No universal best instrument can yet be recommended for all revision pathways.
Study	Selection (0–4)	Comparability (0–2)	Outcome (0–3)	NOS total	Risk-of-bias judgment	Influence on synthesis
Traven et al. [16]	4	2	3	9/9	Low	Large adjusted NSQIP analysis; high weight in consistency assessment.
Meyer et al. [17]	3	1	3	7/9	Moderate	Single-center design and modest sample size reduced certainty.
Raad et al. [18]	4	2	3	9/9	Low	Derivation plus validation strengthened prediction evidence.
Zamanzadeh et al. [19]	4	2	3	9/9	Low	Large aseptic revision cohort supported dose–response inference.
Shi et al. [20]	3	1	2	6/9	Moderate	Small single-center PJI cohort; estimates interpreted cautiously.
Momtaz et al. [21]	4	2	3	9/9	Low	Large adjusted analysis and graded frailty strata strengthened association evidence.
Tram et al. [22]	4	2	2	8/9	Low/moderate	Administrative coding and indication heterogeneity considered.
Kyaw et al. [23]	4	2	2	8/9	Low/moderate	Large NRD cohort but outcome definitions varied by indication.
Arapovic et al. [24]	4	2	2	8/9	Low/moderate	Propensity weighting strengthened inference, but frailty depended on ICD coding.
Pean et al. [25]	3	2	2	7/9	Moderate	Predictive modeling report; incomplete descriptive data limited external interpretation.
Grimmett et al. [26]	4	2	3	9/9	Low	Direct head-to-head comparison in septic revision cohort supported tool-comparison conclusions.

## Data Availability

Not applicable.

## Appendix A. Complete Search Strings and Gray-Literature Procedures

PubMed/MEDLINE: ((“Arthroplasty, Replacement, Hip”[Mesh] OR “Arthroplasty, Replacement, Knee”[Mesh] OR “revision total hip arthroplasty” OR “revision total knee arthroplasty” OR rTHA OR rTKA OR “revision joint replacement” OR “second-stage revision”) AND (“Frailty”[Mesh] OR frail* OR “frailty index” OR “modified frailty index” OR mFI-5 OR mFI-11 OR “hospital frailty risk score” OR HFRS OR “clinical frailty scale” OR CFS OR “CARDE-B” OR “Risk Analysis Index” OR RAI-rev) AND (complication* OR mortality OR readmission OR morbidity OR outcome* OR “length of stay” OR discharge OR reoperation OR reinfection)).
Embase: (‘revision hip arthroplasty’/exp OR ‘revision knee arthroplasty’/exp OR ‘revision total hip arthroplasty’ OR ‘revision total knee arthroplasty’ OR rtha OR rtka OR ‘second-stage revision’) AND (‘frailty’/exp OR frail* OR ‘modified frailty index’ OR ‘hospital frailty risk score’ OR ‘clinical frailty scale’ OR ‘carde-b’ OR ‘risk analysis index’) AND (‘postoperative complication’/exp OR complication* OR mortality OR readmission OR ‘length of stay’ OR discharge OR reoperation OR reinfection).
Cochrane Library: ([revision total hip arthroplasty] OR [revision total knee arthroplasty] OR [revision joint replacement] OR rTHA OR rTKA) AND (frailty OR frail OR modified frailty index OR hospital frailty risk score OR clinical frailty scale OR CARDE-B) AND (complications OR mortality OR readmission OR length of stay OR discharge).
Web of Science Core Collection: TS = ((revision NEAR/2 (hip OR knee OR arthroplasty OR replacement)) OR rTHA OR rTKA OR “second-stage revision”) AND TS = (frailty OR frail* OR “modified frailty index” OR “hospital frailty risk score” OR “clinical frailty scale” OR “CARDE-B” OR “Risk Analysis Index”) AND TS = (complication* OR mortality OR readmission OR “length of stay” OR discharge OR reinfection OR reoperation).
Scopus: TITLE-ABS-KEY((revision W/2 (hip OR knee OR arthroplasty OR replacement)) OR rTHA OR rTKA OR “second-stage revision”) AND TITLE-ABS-KEY(frailty OR frail* OR “modified frailty index” OR “hospital frailty risk score” OR “clinical frailty scale” OR “CARDE-B” OR “Risk Analysis Index”) AND TITLE-ABS-KEY(complication* OR mortality OR readmission OR “length of stay” OR discharge OR reinfection OR reoperation).
Gray literature and conference sources: AAOS, EFORT, and ISAR proceedings were searched for 2019 through January 2026 using the terms revision arthroplasty, revision hip, revision knee, frailty, modified frailty index, hospital frailty risk score, CARDE-B, and risk analysis index. Conference-only abstracts were used for citation tracking only and were not included unless a full-length peer-reviewed article was available.
